# Effects of the Intermittent Pneumatic Circulator on Blood Pressure during Hemodialysis

**DOI:** 10.3390/s101110014

**Published:** 2010-11-09

**Authors:** Tzu-Chao Hsu, Ya-Ju Chang, Yu-Yao Huang, Miao-Ju Hsu

**Affiliations:** 1 Department of Internal Medicine, Pao-Chien Hospital, Ping-Tung City, Taiwan; E-Mail: tc003022@ms34.hinet.net; 2 Physical Therapy Department and Graduate Institute of Rehabilitation Science, Chang Gung University, Tao-Yuan, Taiwan; E-Mail: yjchang@mail.cgu.edu.tw; 3 Department of Cardiology, Pao-Chien Hospital, Ping-Tung City, Taiwan; E-Mail: thinkingteam2000@yahoo.com; 4 Department of Physical Therapy, College of Health Science, Kaohsiung Medical University, Kaohsiung, Taiwan; 5 Department of Rehabilitation, Kaohsiung Medical University Hospital, Kaohsiung, Taiwan

**Keywords:** hemodialysis, dialysis, mechanical pump, blood pressure, circulator

## Abstract

Hypotension is frequently reported during hemodialysis. This study aimed to examine the effect of the intermittent pneumatic circulator on blood pressure during hemodialysis. Sixteen subjects with chronic hemodialysis were recruited. Each subject randomly received two test conditions on separate days, hemodialysis with and without the circulator. The circulator was applied to the subject on lower extremities during 0.5–1 hr, 1.5–2 hr, 2.5–3 hr, and 3.5–4 hr of hemodialysis. Systolic and diastolic blood pressures (SBP and DBP) and heart rate (HR) were analyzed at pre-dialysis, 1 hr, 2 hr, and 3 hr of hemodialysis. Stroke volume (SV) and cardiac output (CO) were evaluated between 2.5 and 3.0 hr of hemodialysis. Blood chemicals (sodium, calcium, potassium, and phosphorous) and Kt/V before and after each hemodialysis session were analyzed. The number of episodes of hypotension was also recorded. The circulator intervention significantly improved SBP and DBP across all time points (P = 0.002 for SBP; P = 0.002 for DBP). The frequency of hypotension was significantly decreased (P = 0.028). SV and CO were significantly improved with the circulator intervention (P = 0.017 for SV; P = 0.026 for CO) and no statistical significances were found on blood chemicals or Kt/V analyses. The results suggested that the circulator intervention helps stabilize blood pressure and appears to be a practical treatment. Future studies are suggested to develop new circulator innovations with sensor feedback systems to enhance safety and maximize treatment efficiency.

## 1. Introduction

Hypotension during hemodialysis is one of the more frequently reported hemodynamic instabilities. The prevalence of a symptomatic reduction in blood pressure during or immediately after hemodialysis ranges from 15 to 50 percent of dialysis sessions [1,2]. In addition to drops in blood pressure, other manifestations may include vomiting, muscle cramps, and other vagal symptoms, such as yawning and drowsiness. Hemodialysis-induced hypotension may prevent uremic patients from a safe and comfortable treatment, reduce treatment efficacy, further decrease residual renal function and increase morbidity related with dialysis procedures [3]. It has been reported that hemodialysis-related hypotension is an independent determinant associated with myocardial stunning [4]. Repetitive myocardial ischemia can be cumulative and may lead to left ventricular dysfunction [5–7], which may further aggravate hypotension during hemodialysis [8,9]. Long-term effects of severe and repeated hypotension may be associated with cardiovascular diseases, such as heart failure, and increase mortality in individuals with hemodialysis [4,10].

The mechanism of hemodialysis-induced hypotension is not fully understood. The determinants of arterial blood pressure are cardiac output and total peripheral vascular resistance. Research has shown that factors affecting cardiac output and/or peripheral resistance, such as diminished cardiac reserve [11], increased synthesis of endogenous vasodilators [12], and failure to increase plasma vasopressin levels [13] contribute to dialysis-related hypotension. Other contributory factors may be related to characteristics of patients, such as age, and dialysis methodology, including rapid fluid removal in an attempt to attain “dry weight” [14], and use of acetate rather than bicarbonate as a dialysate buffer [9,15], *etc*. Among those factors mentioned above, hypovolemia induced by ultrafiltration has been considered the major cause of an acute decrease of blood pressure during hemodialysis treatment [16,17].

The intermittent pneumatic circulator is an electronic mechanical pump commonly used in physical therapy with the main purpose of controlling or reducing edema, prevention of thrombophlebitis, and improving peripheral circulation [17,18]. The circulator intermittently pumps air into an inflatable sleeve or boot where an upper or lower extremity has been inserted. During pumping, the air pressure surrounding the extremity increases and thus the fluids in the interstitial spaces of the extremity are facilitated to return to the venous and lymphatic vessels and then to the heart. Therefore, theoretically, the circulator should be able to improve drops in blood pressure during hemodialysis by increasing preload to the heart. However, no research has been done to test this conjecture. This study aimed to examine the effect of the circulator on blood pressure during hemodialysis. We hypothesized that the circulator would improve drops in blood pressure.

## 2. Methods

### 2.1. Participants and Dialysis Prescription

Sixteen subjects (six males and 10 females) with chronic hemodialysis and with any episode of hypotension during hemodialysis in the past three months were recruited. The hemodialysis-related hypotension was defined as the level of blood pressure low enough to require nursing or medical intervention or drops in systolic blood pressure greater than 30 mmHg [19]. Their age, height, weight, and hemoglobin level were 48.8 ± 9.4 yrs, 158.5 ± 8 cm, 60.7 ± 10.7 kg, and 10.59 ± 1.97 g/dL, respectively. The patients underwent hemodialysis three times weekly, 3.5–4.0 hrs each time. The time of hemodialysis was 8.71 ± 4.06 yrs. The underlying causes of end-stage renal disease were diabetes, chronic glomerulonephritis, and hypertension. Two subjects were taking antihypertensive medications and the dose of medications was kept the same during this study period. On the day of hemodialysis, these two subjects did not take the medications.

The dialysis was performed using either a polysulphone or a polymethylmethacrylate (PMMA) dialyser. The surface membrane area ranged from 1.6 m^2^ to 2.2 m^2^. The ultrafiltration (% body weight reduction) during dialysis sessions was adjusted according to the presumed dry weight (assessed as the post-dialysis patient’s weight when normotensive and free of edema). Dry weight was established by the attending physician. Dry weight and ultrafiltration were kept constant as possible for a specific subject throughout this study.

The subject was afebrile (pre-dialysis temperature 36.3–36.9 °C) and the temperature of the dialysate was kept constant at 37 °C. The blood flow and dialysate flow rates were 250–300 mL/min and 500 mL/min, respectively. The dialysate chloride, bicarbonate, calcium, sodium, potassium, and glucose concentrations were 106.5–107.5 mEq/L, 39 mEq/L, 2.5–3.5 mEq/L, 138–141 mEq/L, 2.0 mEq/L, and 200 mg/dL, respectively.

### 2.2. Experimental Procedure

Written informed consent was obtained from the subject before enrollment in this study. Study protocols were conducted in accordance with the ethical guidelines of Kaohsiung Medical University. Each subject randomly received two test sessions exactly one week apart, hemodialysis with and without the circulator. For each test session, blood pressure (BP) and heart rate (HR) were taken every 30 minutes with mercury sphygmomanometer and heart rate monitor (CheckMyHeart; DailyCare, Taipei, Taiwan) and the data at pre-dialysis, and 1 hr, 2 hr, and 3 hr of hemodialysis were analyzed. Stroke volume (SV) and cardiac output (CO) were evaluated by Doppler echocardiography (Philips SONOS 5500; Philips, Seattle, WA, USA) in supine position between 2.5 and 3.0 hr of hemodialysis. SV was calculated by the following equation: SV = LVOT^2^ × 0.785 × VTI, where LVOT and VTI were left ventricle outflow tract and velocity-time integral, respectively. Blood was sampled before and after hemodialysis session and analyzed with a model 200FR automatic analyzer (Toshiba 200; Toshiba, Tokyo, Japan) for sodium, potassium, calcium, phosphorous, and blood urea (expressed as BUN) concentrations. The number of episodes of hypotension during hemodialysis that required clinical intervention was also recorded. Delivered dose of dialysis (Kt/V, where K, t, and V are the clearance (m^3^/s), time (s), and the distribution volume of urea (m^3^), respectively) was calculated according to National Kidney Foundation Disease Outcomes Quality Initiative (NKF-DOQI) recommendation.

**Figure 1.**
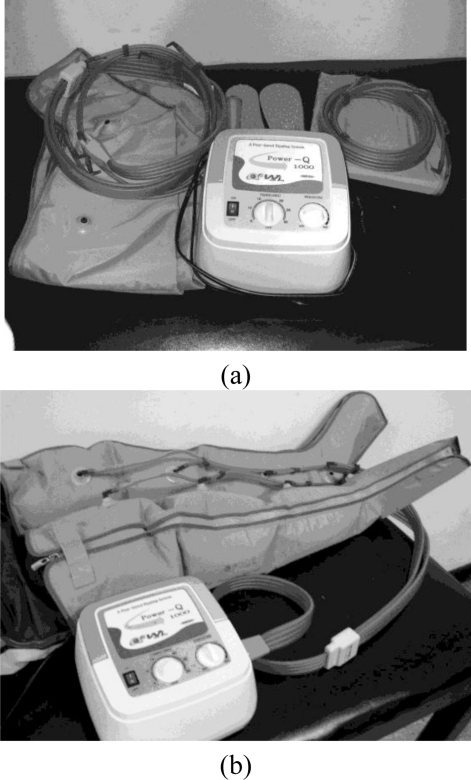
**(a)** The circulator and its accessories; **(b)** The application of the circulator.

For the test session with the circulator intervention, the circulator (Power Q1000; Wonjin Mulsan Co., Ltd., Incheon, Korea) was applied to the subject on both lower extremities (Figure 1) for half an hour at 0.5 hr, 1.5 hr, 2.5 hr, and 3.5 hr of hemodialysis, with a total intervention time of two hours. The leg cuffs of the circulator were gradually inflated from distal to proximal (Figure 1). The pressure of pumping was determined by the comfort level of the subject, but was less than the subject’s diastolic blood pressure at pre-dialysis.

### 2.3. Data Analysis

Dependent variables include systolic blood pressure (SBP), diastolic blood pressure (DBP), HR, SV, CO, Kt/V, blood chemicals (sodium, calcium, potassium, and phosphorous), the number of episodes of hypotension, and ultrafiltration volume. Descriptive statistics was used to analyze mean and standard deviation for each variable. SBP, DBP, and HR were expressed as a percentage of the baseline (pre-dialysis) SBP, DBP, and HR, respectively. The two-way repeated measures ANOVA was used to analyze the differences on SBP, DBP, and HR between hemodialysis with and without the circulator intervention. Tukey-Kramer test was employed for post-hoc analysis when appropriate. The paired-t test was used to analyze the differences on SV, CO, Kt/V, blood chemicals, the frequency of hypotension, and ultrafiltration volume. A significant level was set at 0.05.

## 3. Results

**Figure 2.**
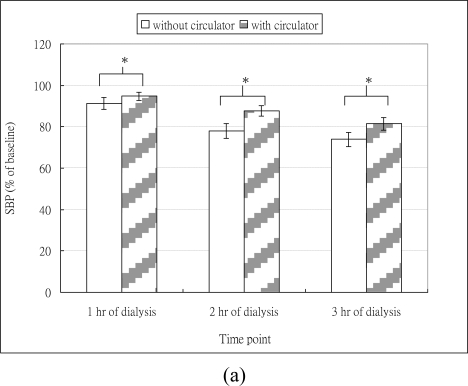

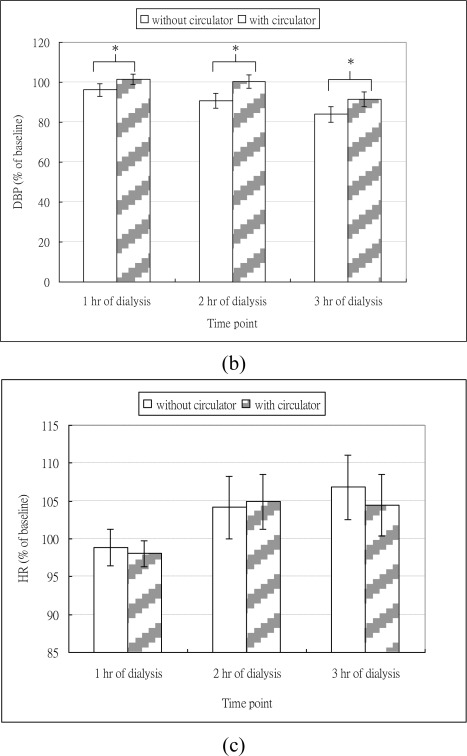
Mean and standard errors of SBP, DBP, and HR at different time points: **(a)** SBP; **(b)** DBP; **(c)** HR. (*A significant level was set at 0.05).

The mean and standard deviation of SBP, DBP and HR at different time points are shown in Table 1. As shown in Figure 2(a), regardless of whether dialysis was with or without circulator, SBP decreased at different time points. The ANONA results showed significant time effects on SBP (Table 2). The *post-hoc* test revealed that compared to baseline, SBP at 1 hr (P = 0.010), 2 hr (P < 0.001), and 3 hr (P < 0.001) of hemodialysis were significantly decreased. As shown in Figure 2(b), for dialysis without circulator, DBP decreased across different time points and the extent of the decreased DBP appeared to be aggravated with the increase of time. For dialysis with circulator, DBP slightly increased at 1 hr, but decreased at 3 hr time point. The ANOVA results revealed that significant time effects on the DBP (Table 2). However, the *post-hoc* analysis showed that only the DBP at 3 hr (P < 0.001) significantly decreased, compared to the baseline. These results indicated hypotension occurred in our subjects during hemodialysis.

As for the intervention effects of the circulator, the ANOVA revealed that dialysis with circulator significantly attenuated the drops in SBP and DBP across different time points (Table 2). The number of episodes of hypotension for hemodialysis with and without the circulator intervention was 1.56 ± 0.96 and 0.94 ± 0.77 respectively, and the paired-t revealed significantly decreased episodes for dialysis with the circulator (P = 0.028). These results supported our hypothesis that the circulator would improve blood pressure during hemodialysis.

As shown in Figure 2(c), regardless of whether dialysis was with or without circulator, the HR decreased at 1 hr but increased afterwards. The ANOVA results showed significant time effects on HR. However, the *post-hoc* test revealed that compared to the HR at baseline, no significant differences were found at all time points. The ANOVA results showed no effect of circulator intervention on HR.

Two subjects refused the Doppler echocardiography examination. The mean and standard deviation (N = 14) for SV with and without circulator were 58.80 ± 18.62 L/min and 45.16 ± 17.71 mL/min, respectively. HR with and without circulator were 88.4 ± 18.9 bpm and 89.7 ± 16.8 bpm, respectively. CO with and without circulator was 5.06 ± 1.51 L/min and 3.98 ± 1.50 L/min, respectively. The paired-t test showed that compared to dialysis without circulator, both the SV (P = 0.017) and the CO (P = 0.026) were significantly increased with the circulator intervention, while no difference in HR (P = 0.551).

The Kt/V, indicating the efficiency of hemodialysis, was 1.38 ± 0.37 for dialysis with circulator and 1.36 ± 0.34 for dialysis without circulator. No significant difference for Kt/V between dialysis with and without circulator was found (P = 0.245). The mean and standard deviation for sodium, calcium, potassium, and phosphorous are presented in Table 3. No significant differences were found between dialysis with and without circulator (P = 0.894 for sodium, P = 0.370 for calcium, P = 0.683 for potassium, and P = 0.744 for phosphorous). The mean values for ultrafiltration volume with and without circulator were 3.51 ± 0.97 kg and 3.39 ± 1.18 kg, respectively. The paired-t test revealed that no significant differences (P = 0.39) on fluid removal were found between two test sessions.

## 4. Discussion

Hypotension is one of the most common complications seen during hemodialysis. Repeated severe symptomatic hypotension might result in brain and cardiac tissue damage, and correlates with long-term cardiovascular problems [4,20]. Previous studies reported up to 50% drop in SBP [21,22]. In our study, the amount of decreased SBP during dialysis without circulator intervention ranged from 9% (1 hr) up to 27% (3 hr), which appears to be at the lower end of the range reported. We recruited hypotension-prone subjects, while some research had more severe inclusion criteria for subject selection, such as at least a 25% drop in SBP. Recruitment criteria/characteristics for subjects might contribute to the wide range of hemodialysis hypotension seen in the literature. Gender differences on hemodialysis-related hypotension are controversial [2]. We further analyzed our data and found no significant gender differences on reductions in SBP across all time points, though female and male subjects had up to 28% and 23% drops in SBP respectively.

The intermittent pneumatic pressure pump or circulator is usually used in physical therapy to facilitate peripheral circulation. It is speculated to be able to improve drops in blood pressure during hemodialysis by increasing venous return to the heart, according to its theoretical beneficial effects. However, to our knowledge, this is the first study to substantiate this conjecture. In our study, though the SBP during hemodialysis with circulator was still dropped but the extent of the decreased SBP was significantly improved from 9% to 5% at 1 hr and 27% to 19% at 3 hr. In addition, the number of episodes of hypotension that required clinical intervention was significantly reduced with the circulator intervention (P = 0.028). It is noteworthy that the circulator intervention may be also effective for the hemodialysis patient with chronic hypotension defined as SBP less than 100 mmHg in the interdialytic period [23,24]. One of our subjects had chronic hypotension and appeared to respond well to the intervention. Without the circulator intervention, SBP during hemodialysis was gradually dropped from 3% at 1 hr to 15% at 3 hr, while SBP under the circulator intervention condition did not drop at the first two hours and only showed a drop of 10% at 3 hr.

Adequate blood volume is important to maintain a stable blood pressure. Hypovolemia is considered a main factor contributing to dialysis-related hypotension [17]. Poldermans *et al.* investigated the presence of myocardial ischemia and myocardial contractile reserve during infusions of the β-adrenergic receptor agonist dobutamine in hypotension-prone and hypotension-resistant hemodialysis patients and proposed that hemodialysis-induced hypovolemia caused a fall in cardiac filling pressure and thus decrease in CO. Consequently, sympathetic nervous system was activated to adequately increase CO. If not, hypotension happened. This proposed mechanism has been evidenced by studies finding that drops in blood pressure were often paralleled by reductions in blood volume expressed by a percentage of the starting blood pressure [25,26]. In our study, with circulator, an increase of 27.1% in CO was found and this might significantly contribute to more stable SBP seen during dialysis with circulator. However, due to equipment accessibility and subject compliance, we did not measure CO for multiple time points. Therefore, we won’t be able to examine the relationship between CO and decreased blood pressure with time.

Normal compensatory cardiac strategies to prevent hypovolemia are to increase in HR and contractility. However, an increase in HR, caused by beta-adrenergic activation, is of relatively minor importance in maintaining blood pressure [27]. Thus, diminished ability to increase contractility will cause increased sensitivity to hypovolemia, which is especially true for dialysis patients with left ventricular diastolic dysfunction. In case of extreme intracardial hypovolemia, in order to prevent myocardial damage, a cardio-inhibitory reflex might be induced and results in bradycardia and further aggravate hypotension [28]. Obviously, this is not the case in our study. Our subjects demonstrated that compared to baseline, small reductions in HR at the first hour of hemodialysis session but HR increased later on (Figure 2(c)). The initial drops in HR might be due to the decrease in blood volume caused by ultrafiltration and the increased HR afterwards might result from an activation of baroreflex as a compensatory strategy for hypovolemia. However, even though there were fluctuations in HR responses, no significant differences across time points were found, suggesting HR may be a minor contributory factor to prevent hemodialysis hypotension, as mentioned above. This is further evidenced by our data from echocardiograpy, which showed that during hemodialysis with circulator, CO was predominantly improved as a result of significantly increased SV, instead of HR.

Blood pressure is determined by CO and total peripheral resistance. In our study, we were unable to utilize a doppler echocardiography to continuously monitor CO during hemodialysis. Therefore, we did not know if CO changes paralleled with drops in blood pressure throughout dialysis process. Previous studies suggested impaired baroreflex sensitivity and arteriovenous tone adjustment to hypovolemia might be associated with hemodialysis-induced hypotension [29,30]. It is unknown if those factors contributed to drops in blood pressure of our subjects, due to lack of measurements on baroreflex sensitivity and total peripheral resistance in this study.

The Kt/V, and other blood chemical analyses (sodium, potassium, calcium, phosphorous) were not significant, indicating the circulator intervention had no influence on clearance of blood chemicals. The Kt/V is a multiple of the volume of plasma cleared of urea divided by the distribution volume of urea and has been considered an indicator of hemodialysis efficiency. An increase in CO possibly facilitates the clearance of urea during dialysis. However, in our study, the circulator intervention appeared not to enhance the efficiency of hemodialysis, though a significant increase in CO was found. Nevertheless, one should note that our subjects only received the circulator intervention for a total of two hours during a 3.5 to 4.0 hour dialysis session. The influence of the circulator intervention for a longer period of time on the efficiency of hemodialysis deserves more investigations. In addition, a small sample size of 16 may limit the power for the circulator intervention to show significant differences on Kt/V. A post-hoc power analysis using a two-tail significant test revealed that the effect size of the circulator intervention on Kt/V was only 0.33. A larger sample size is suggested for the future study to achieve statistical power.

Kyperkalemia rebound after hemodialysis, *i.e.*, the increment of plasma potassium detectable within the initial few hours after dialysis, may potentially cause fatal cardiac arrhythmia, and enhanced muscle weakness and fatigue [31,32]. It is associated with redistribution of potassium between intra-/extracellular compartments post hemodialysis [33]. Kong *et al*. found exercise during hemodialysis could improve hyperkalemia rebound. The underlying mechanism governing this improvement was suggested to be related to the increase of plasma concentration of potassium as a result of efflux from the contracting muscles and thus, the potassium removal during hemodialysis was enhanced [34]. In our study, the circulator intervention mimic massage movements on lower extremities of the subject [35], which may potentially aid in potassium removal during hemodialysis.

Several types of pneumatic circulators are available on market. The circulator used in this study provided a gradient design, which was designed to incorporate the massage effect of a distal to proximal pressure with a gradual decrease in the pressure gradient. Some other types of intermittent circulator can control timing of pumping during appropriate heart cycle, such as a more advanced innovation, the end-diastolic pneumatic boot. Dillon *et al*. found the increases in CO and SV were greater for pumping during end-diastolic phase than during systolic phase while afterload was much increased for pumping during systolic phase [36]. Timing of pumping appears to affect the preload and afterload of the heart and thus influences CO. Coupling between the cardiac cycle and pumping may enhance the venous return and should be taken into account when developing new circulators. Furthermore, clinical research is warrant to investigate whether these new circulators with sensors to control the timing of pumping provide better improvement on hemodialysis hypotension.

Intermittent pneumatic circulator might expel arterial blood from the legs, potentially worsening peripheral ischemia and thus inducing muscle cramps and increasing discomfort, which is especially true in higher compression pressure settings. A pressure approximating the patient’s DBP has been suggested to be used in most treatment protocols [37]. Our pressure setting complied with this principle. Though a couple of our subjects complained of muscle cramps, all of them had had prior history of muscle cramps before entry of this study. Namely, the circulator intervention did not induce higher incidence of muscle cramps during hemodialysis. In addition, no patients reported the increase of discomfort for using the circulator. It appears that the application of the circulator during hemodialysis is practical. However, as high compression pressure might cause tissue ischemia, a circulator will provide a safer and more efficient intervention if it has a sensor control feedback system which can monitor the subject’s DBP throughout the circulator intervention and automatically adjust the pumping pressure to the appropriate level. This type of the circulator would be particularly beneficial in long-term use for the subject whose DBP may fluctuate during the circulator intervention, such as individuals with hemodialysis or instable cardiac hemodynamics.

Basically, the circulator intervention improves drops in blood pressure during hemodialysis through the mechanism of increasing venous return. Other interventions based on the similar principal may be also useful to prevent hemodialysis-related hypotension, such as abdominal compression. Abdominal compression has been shown to help overcome orthostatic hypotension [38–40], including post-dialytic orthostatic hypotension [38]. Therefore, it may also aid in improving hypotension during hemodialysis, though to our knowledge, evidence in this case has not been reported.

In summary, even though hypotension still occurred during hemodialysis, the degree of the decreased blood pressure was significantly improved with the circulator intervention by means of increasing preload to the heart. Furthermore, the frequency of hypotension during hemodialysis was significantly decreased. The circulator intervention was evidenced to help stabilize blood pressure. In our study, the total treatment time of the circulator intervention was only about 50% of a hemodialysis session. In addition, the circulator used in this study was unable to pump in accordance with heart cycles, nor adjust compression pressure to cope with the subject’s DBP. These limitations of this study might minimize the extent of improvement seen in the circulator intervention. Developing more advanced circulators with sensor feedback systems is recommended and would enhance the application of the circulator intervention.

## 5. Conclusions and Clinical Application

Hypotension during hemodialysis is a common reported complication. Intermittent pneumatic circulator can attenuate drops in blood pressures and appears to be a practical intervention. Future studies are suggested to develop new circulator innovations with sensor feedback systems to enhance safety and maximize treatment efficiency.

## Figures and Tables

**Table 1.** Mean and standard deviation of SBP, DBP, and HR at different time points.

**Without circulator**				

	Baseline	1 hr of dialysis	2 hr of dialysis	3 hr of dialysis
SBP (mmHg)	151.8 ± 29.2	138.1 ± 30.9	116.3 ± 21.6	109.9 ± 21.2
DBP (mmHg)	78.6 ± 12.2	75.3 ± 13.5	71.2 ± 16.0	65.3 ± 13.0
HR (bpm)	86.3 ± 11.0	85.3 ± 11.2	89.8 ± 15.6	92.0 ± 15.3

**With circulator**				

	Baseline	1 hr of dialysis	2 hr of dialysis	3 hr of dialysis

SBP (mmHg)	146.5 ± 28.0	137.4 ± 20.0	126.6 ± 19.2	114.9 ± 16.7
DBP (mmHg)	79.1 ± 12.3	79.6 ± 10.3	78.4 ± 8.7	70.4 ± 8.8
HR (bpm)	84.5 ± 10.4	83.1 ± 13.1	88.6 ± 15.2	88.1 ± 17.6

**Table 2.** The two-way (with/without circulator by time points) ANOVA summary.

	**SBP**		**DBP**		**HR**	

	F	P	F	P	F	P
With/without circulator	10.15	0.002^*^	9.73	0.002^*^	0.09	0.769
Time point	41.66	<0.001^*^	9.87	<0.001^*^	4.78	0.004^*^
Interaction	1.72	0.168	1.34	0.266	0.10	0.960

* A significant level at 0.05.

**Table 3.** Mean and standard deviation of sodium, calcium, potassium, and phosphorous.

	**Without circulator**			
	Sodium (meq/L)	Calcium(mg/dl)	Potassium(meq/L)	Phosphorous(meq/L)
Before dialysis	135.94 ± 3.99	9.56 ± 1.19	4.49 ± 0.90	5.14 ± 1.72
After dialysis	138.27 ± 3.08	10.55 ± 0.73	3.55 ± 1.00	2.31 ± 1.15

	**With circulator**			
	Sodium (meq/L)	Calcium(mg/dl)	Potassium(meq/L)	Phosphorous(meq/L)

Before dialysis	135.94 ± 3.36	9.79 ± 0.22	4.44 ± 1.06	5.18 ± 1.96
After dialysis	138.19 ± 3.73	10.39 ± 1.13	3.69 ± 0.94	2.55 ± 1.25
